# Progress in the discovery of isopods (Crustacea: Peracarida)—is the description rate slowing down?

**DOI:** 10.7717/peerj.15984

**Published:** 2023-09-04

**Authors:** Lena Hartebrodt, Simon Wilson, Mark John Costello

**Affiliations:** 1 Institute of Marine Science, University of Auckland, Auckland, New Zealand; 2 School of Computer Science and Statistics, University of Dublin, Trinity College, Dublin, Ireland; 3 Faculty of Biosciences and Aquaculture, Nord University, Bodø, Norway

**Keywords:** Biodiversity, Isopoda, Description rate, Taxonomic effort

## Abstract

Taxonomic species are the best standardised metric of biodiversity. Therefore, there is broad scientific and public interest in how many species have already been named and how many more may exist. Crustaceans comprise about 6% of all named animal species and isopods about 15% of all crustaceans. Here, we review progress in the naming of isopods in relation to the number of people describing new species and estimate how many more species may yet be named by 2050 and 2100, respectively. In over two and a half centuries of discovery, 10,687 isopod species in 1,557 genera and 141 families have been described by 755 first authors. The number of authors has increased over time, especially since the 1950s, indicating increasing effort in the description of new species. Despite that the average number of species described per first author has declined since the 1910s, and the description rate has slowed down over the recent decades. Authors’ publication lifetimes did not change considerably over time, and there was a distinct shift towards multi-authored publications in recent decades. Estimates from a non-homogeneous renewal process model predict that an additional 660 isopod species will be described by 2100, assuming that the rate of description continues at its current pace.

## Introduction

Species richness is a commonly used metric to measure biodiversity. Knowing how many different species there are in space and time is vital for all biodiversity-based research and sustainable conservation strategies. Scientists have long tried to answer the intriguing question of how many species exist on Earth. Estimates range from about 2 million species (Costello, Wilson & Houlding, 2012) to 10 million species (Grassle & Maciolek, 1992). Even numbers from “at least 1 billion to 6 billion” species have been estimated based on various assumptions like parasite-host ratios and a very high ratio of bacterial to animal species (Larsen et al., 2017). Many recent estimates of total species richness for different taxa are based on observed description rates, often from a global dataset which buffers local biases, and are of a more conservative nature (*e.g.*, Bebber et al., 2007; Costello, 2016; Deng et al., 2016).

The first question to ask when it comes to estimating total species richness is how many species have already been described. At the beginning of this century this question was still difficult to answer. Compiling global datasets for various taxa would have been very time-consuming and tedious. The bulk of knowledge in the field of taxonomy was hidden away in large and expensive printed monographs or low-impact and regionally restricted print-only journals that could be hard to come by (Godfray, 2002). Godfray (2002) also stated that “taxonomy is made for the web” and needs to reinvent itself “as a twenty-first-century information science” where the global knowledge and achievements of the field are collected in one place and made easily accessible for everyone. Now, with the publication of continually updated databases like the Catalogue of Life (Bánki et al., 2021) and the World Register of Marine Species (Ahyong et al., 2023), which also account for some known synonymies, assessing the number of already described species is a lot easier, and many studies make use of these data (*e.g.*, Mora et al., 2011; Costello, Wilson & Houlding, 2012; Arfianti, Wilson & Costello, 2018; Pamungkas et al., 2019; Pagès-Escolà et al., 2020).

The rate of description of new species also depends on the number of taxonomists working towards a complete inventory of life on Earth. Some studies raised concerns that taxonomy was in crisis (Gaston & May, 1992; Hopkins & Freckleton, 2002; Bacher, 2012). While this may be true in some institutions and for some taxa, it does not apply to the global workforce. Other studies found that, in contrast to a proposed decline in the taxonomic workforce, the number of people describing new species has been increasing over recent decades (*e.g.*, Eschmeyer et al., 2010; Appeltans et al., 2012; Costello, Wilson & Houlding, 2013; Arfianti, Wilson & Costello, 2018; Songvorawit, Quicke & Butcher, 2021). However, the average number of species described per taxonomist showed a decrease (Costello, Wilson & Houlding, 2012), sometimes interpreted as a sign that it is getting harder to find new species from the shrinking pool of still undescribed species (Joppa, Roberts & Pimm, 2011b).

Isopods are a species-rich taxon of crustaceans found globally in terrestrial, marine and freshwater habitats. Based on expert opinion, Isopoda were said to be a promising taxon for tens of thousands of new species (Appeltans et al., 2012). Nevertheless, Poore & Bruce (2012) noted that the description rate of non-asellote marine isopods has slowed down since the 1990s. In a review by Williams & Boyko (2012) it was briefly mentioned that descriptions for parasitic isopods from the superfamilies Bopyroidea and Cryptoniscoidea (which were excluded from Poore & Bruce, 2012) showed two apparent peaks during the 1880–1930s and 1980–2000, while Costello (2016) found that the rate of description of parasitic isopods overall was declining since the 1990s. Previously, Costello, Wilson & Houlding (2012) tried to predict the number of yet undescribed marine isopods based on past description rates. However, their statistical model yielded high uncertainties because the accumulation curve of species numbers still showed a steep increase and was not yet nearing an asymptote. Since these studies, many more species names have been added to WoRMS and more synonymies have been resolved. With this matured dataset of isopods available, this study examines the description rate for the whole order Isopoda, including terrestrial, marine and freshwater species, and subsets of parasitic and subterranean species. Moreover, an estimate of still undescribed isopod species is calculated by the non-homogeneous renewal process (NHRP) model after Wilson & Costello (2005). The NHRP is designed for this purpose and takes into account the variation between years to produce confidence limits around its predictions (Wilson & Costello, 2005). Additionally, indicators of taxonomic effort, such as the number of authors describing species, potentially biased by varying publication lifetimes of authors over time and changing trends in authorship practices, were analysed.

## Methods

Data including species names, authorities, the year of description and environment for the order Isopoda Latreille, 1,816 were downloaded from the World Register of Marine Species (WoRMS) on 19th July 2018 and updated on 20th February 2023 (WoRMS, 2023) after a delay due to the COVID-19 pandemic. All results, figures and tables in this manuscript refer to the updated 2023 dataset. During the cleaning process of the update some substantial changes to the taxonomy of bopyroid and cryptoniscoid isopods, addressed in Boyko & Williams (2023), came to our attention and were incorporated into the update. Although WoRMS is predominantly a database for species that occur in marine habitats, it contains sub-registers like the World Marine, Freshwater and Terrestrial Isopod Crustaceans database. Therefore it was possible to extract data not only for marine isopods but also for freshwater and terrestrial species, allowing an analysis of the whole order Isopoda. To avoid overestimating the actual global number of isopod species, only species names listed in WoRMS as “accepted” and checked by a taxonomic editor have been included in the analysis. Moreover, only extant species and those ranked as “species” were analysed, excluding more than 30 fossil isopods and more than 500 subspecies, though their status was “accepted”. That left a species list with 10,333 entries for the 2018 dataset (Hartebrodt, 2019) and 10,687 accepted species in the updated list from 2023 (Hartebrodt, 2023).

The data were checked for issues that may affect the analysis, and uncertainties were double-checked with WoRMS and corrected. The most common issues were misspellings and different spellings of authors’ surnames like “Magniez” and “Magneiz/Magnez/Magiez” or “Wägele” and “Waegele”. Those were corrected and only one spelling for each surname was used. In cases where different authors had the same surname, it was checked back with the original species descriptions to sort out individual authors. They were distinguished by adding their given names’ initials (*i.e.,* E.H. Williams, J.D. Williams, and W.D. Williams). The number of taxonomists describing species over time is an indicator of taxonomic effort, which could be biased by changing authorship practices (Joppa, Roberts & Pimm, 2011a; Essl et al., 2013; Costello, Wilson & Houlding, 2013; Fisher et al., 2018). For the purpose of this paper, every author who published a scientific description of an isopod species is termed a “taxonomist” without any regard for the extent of his/her expertise in isopod taxonomy. In this analysis, only first authors have been considered to provide a minimum estimate of effort.

Isopods were classified as marine, freshwater or terrestrial species according to the environmental information available in WoRMS. Species inhabiting brackish environments were grouped with the marine species. In addition, subgroups of parasitic and subterranean isopods were classified from the literature. Only isopods that are obligate parasites were classified as “parasitic”. Therefore, species of Corallanidae and Aegidae, often termed as parasites, were not included since those are micropredators (Brusca, 1983) rather than parasites by definition. In the subterranean category, stygobionts and troglobionts were included but not stygophile or troglophile isopod species because these usually have populations that live entirely aboveground.

The data were analysed in several ways to get an accurate picture of the description rate of isopod species over time. First, the cumulative number of species described per year was plotted to see whether there was a levelling out in recent years. Second, the annual number of species’ descriptions was plotted to investigate the general trend of the description rate. Additionally, the non-homogeneous renewal process (NHRP) model of Wilson & Costello (2005) was used to make predictions about future discoveries. The model not only extrapolates the rate of description but also takes into account that description rates differ over time. It was used to estimate numbers on how many isopod species might be described by the years 2050 and 2100 with a 95% confidence interval. The equation used by the NHRP model is the following:

\begin{eqnarray*}t= \frac{N}{1+\exp \nolimits (-\beta \left( t-\alpha \right) )} \end{eqnarray*}

Here *t* is the number of isopod species described by a particular year; *N* is the total number of species to be described; *β* stands for the overall rate of description, and *α* is the year of the maximum rate of description. A larger *β* implies a faster rate of description.

To estimate taxonomic effort, the number of first authors per year was plotted. Furthermore, the average number of species described per number of authors in a year was analysed over time. To determine the breakpoint from whereon the yearly average number of species described per author started to decline, a piecewise regression analysis was performed in R using the “Segmented” package (Muggeo, 2008).

The publication lifetime of first authors was calculated as the number of years from an author’s first description of an isopod species to their most recent. Decreasing lengths of publication lifetimes might suggest a decrease of taxonomists specialised in isopods. To examine whether there was a change in the span of authors’ publication lifetime, linear regressions of publication lifetime against the year of an author’s first species description were performed. Also, linear regressions on publication lifetime against the average number of species described by each author per year were performed to examine whether it has a significant effect on productivity. The regressions were done for all authors, once including and once excluding Vanhöffen, who published the descriptions of all 67 species he described in one extensive monograph resulting in a publication lifetime of only one year.

Authorship practices change over time and might bias the overall estimate of taxonomic effort. Over the years, there might be a trend toward multi-authored species descriptions, termed the “et al.” effect. During the analysis, the number of descriptions with multiple authors was counted, as well as the number of descriptions that had only a single author. Both were plotted per decade to compare them. The number of one-time authors, who described only a single isopod species, was also counted and was plotted as a proportion of all species descriptions per decade.

## Results

### Species diversity

Between 1758 and 2023 a total of 10,687 extant isopod species have been described by a cohort of 1,144 authors (755 first authors). Of the first authors analysed here, 282 were one-time authors who described only a single isopod species. The 21 most prolific authors, each describing more than a hundred species, together described about 43% of all accepted species (see Table S1). More than half of all named species are marine species—6,151 in number. Isopods are the most species-rich crustaceans on land, with 3,840 terrestrial isopod species and 696 freshwater species. Approximately 14% of all species are obligate parasites, and 9% can be categorised as subterranean (*i.e.,* cave-dwellers, groundwater species, inhabitants of interstitial spaces). The order Isopoda consists of 12 suborders comprising 141 families and 1,557 genera. The most species-rich genera, each containing over 100 species, are *Porcellio*, *Armadillidium, Cirolana*, *Gnathia*, *Venezillo*, *Proasellus* and *Trichoniscus*. The most species-rich isopod families are Sphaeromatidae, Armadillidae and Bopyridae (Table 1). At the other end of species richness, there are 15 monotypic families, which have only one genus containing a single species.

**Table 1:** A list of the 32 most species-rich families, each with more than 100 species. Families are ranked by the number of species. The percentage of species described within a family by certain time points is given.

**Family**	**# genera**	**# species**	**First species described**	**Last species described**	**% of species described by**			
					1850	1900	1950	2000
Sphaeromatidae	100	664	1787	2021	6	17	45	89
Armadillidae	82	647	1816	2023	1	14	60	94
Bopyridae	170	639	1798	2023	1	9	46	83
Cirolanidae	63	525	1804	2023	1	10	25	77
Trichoniscidae	87	524	1818	2023	1	4	46	89
Philosciidae	112	508	1763	2023	1	5	27	83
Cymothoidae	45	384	1758	2023	9	38	50	85
Munnopsidae	43	342	1861	2022	0	11	26	81
Asellidae	19	333	1758	2022	1	5	26	90
Porcellionidae	19	330	1804	2023	7	35	72	96
Anthuridae	26	309	1808	2022	1	3	12	89
Armadillidiidae	18	272	1798	2023	5	19	58	84
Eubelidae	50	257	1873	2018	0	14	53	94
Gnathiidae	12	237	1804	2023	1	8	32	72
Idoteidae	24	190	1766	2017	13	32	57	93
Agnaridae	14	189	1771	2022	3	9	44	77
Paramunnidae	45	185	1864	2022	0	4	21	46
Janiridae	22	175	1814	2022	2	11	38	94
Arcturidae	14	161	1806	2021	2	14	48	83
Aegidae	8	149	1758	2023	8	35	56	73
Desmosomatidae	20	145	1864	2020	0	6	19	75
Platyarthridae	8	136	1833	2021	1	9	44	83
Haploniscidae	8	125	1877	2017	0	1	7	74
Trachelipodidae	8	125	1833	2017	3	16	56	90
Styloniscidae	17	124	1853	2022	0	4	35	69
Munnidae	6	114	1839	2023	3	10	36	91
Ligiidae	6	113	1767	2022	7	20	54	78
Ischnomesidae	9	109	1866	2019	0	6	22	80
Scleropactidae	26	108	1854	2021	0	10	34	77
Serolidae	22	107	1775	2015	4	21	36	80
Antarcturidae	18	106	1881	2022	0	10	34	89
Leptanthuridae	14	105	1853	2021	0	9	21	93

**Figure 1:**
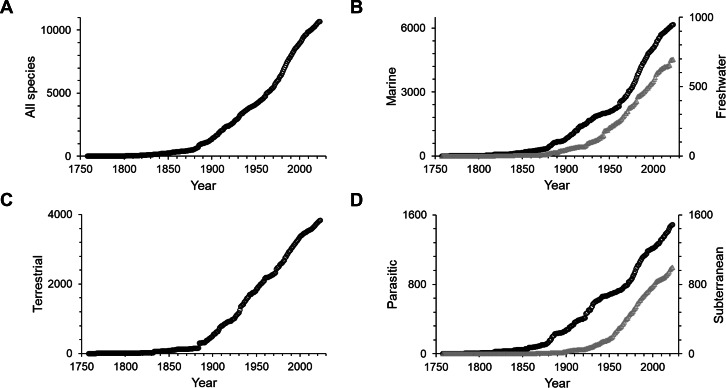
Cumulative numbers of isopod species described per year. (A) All isopods, (B) marine (black circles) and freshwater (grey triangles), (C) terrestrial and (D) parasitic (black circles) and subterranean (grey triangles). Note that the scales vary.

**Figure 2:**
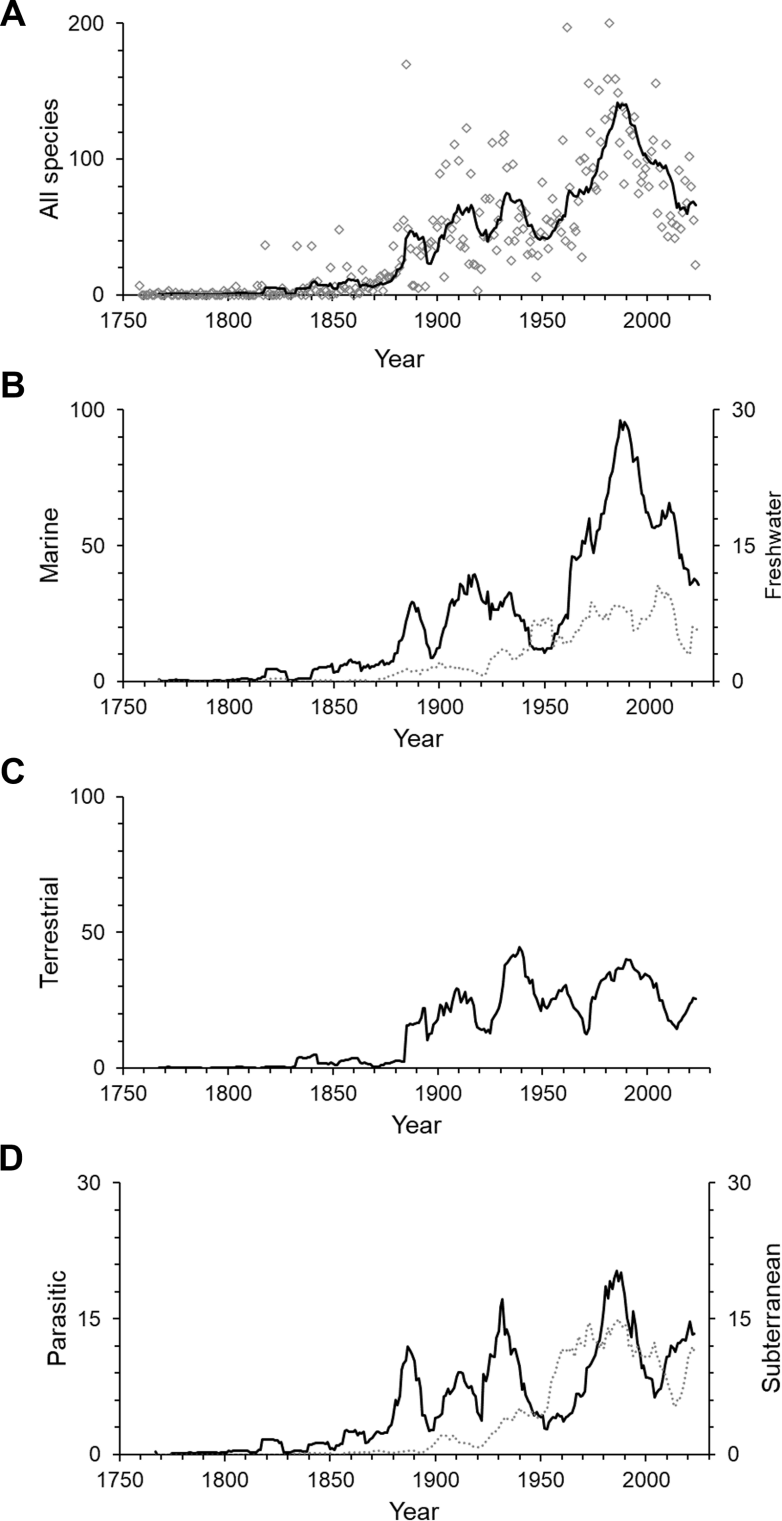
The number of isopod species described per year. (A) All isopods, (B) marine (solid line) and freshwater (dotted line), (C) terrestrial and (D) parasitic (solid line) and subterranean (dotted line). The lines are 10-year moving averages. Note that the scales vary.

The first 100 years of discovery after the publication of Linnaeus’ *Systema Naturae* in 1758, in which the first seven still valid isopod species were described, yielded relatively few species. Until the end of the 18th century an average of only 6 species were described per decade. The following 50 years saw, on average, 43 species descriptions per decade, many of which were contributed by the three most prolific taxonomists of that time. Leach described 30 species between 1814 and 1818; J.F. Brandt contributed 37 species descriptions between 1831 and 1841; and H. Milne-Edwards added 34 new species in 1840, at which point the overall number of named isopod species had climbed to 194. For a detailed history of the discovery of marine isopods see Poore & Bruce (2012). From the 1850s to the end of the 19th century the average number of new species per decade climbed to 209. Descriptions of new isopod species started to accumulate faster, and after the 1880s the rate increased swiftly and steadily up to the 1970s, when the slope of the curve got even steeper (Fig. 1A). The terrestrial subgroup follows this overall pattern very closely (Fig. 1C), whereas for marine isopods the cumulative number of species seemed to plateau for short periods of time in the 1890s and the mid-20th century, before resuming a steep increase after the 1960s (Fig. 1B). A dip in descriptions during World War II and its aftermath is clearly visible in almost all groups (Figs. 2A–2D). Only freshwater isopods show a small peak in species descriptions during that time, largely due to Nicholls’ work, who published 36 descriptions of freshwater isopods in 1943 and 1944 (Fig. 2B). Besides having far lower species numbers than marine isopods, discoveries of freshwater species stayed low until the 1880s (Fig. 1B). The discovery of subterranean species started later, and most were discovered after the 1950s (Fig. 1D).

Isopods showed a peak of discovery in the late 20th century, with an all-time high of 200 species described in the year 1982 (Fig. 2A). Most subgroups peaked during the same period, except for freshwater isopods, which had their highest peak at the beginning of the 21st century and terrestrial species having their main peak earlier in the 1930s (Figs. 2B–2D). In the past three decades the number of species described per year has decreased notably in overall species descriptions and specifically marine isopods. Yearly descriptions of freshwater isopods are generally low, although 2020 was a record year that saw 34 freshwater species described. This was more than 10-times the average of the previous 10 years. On average one third of yearly descriptions over the past 10 years were parasitic and subterranean species.

### Predictions of yet to be named species

**Figure 3:**
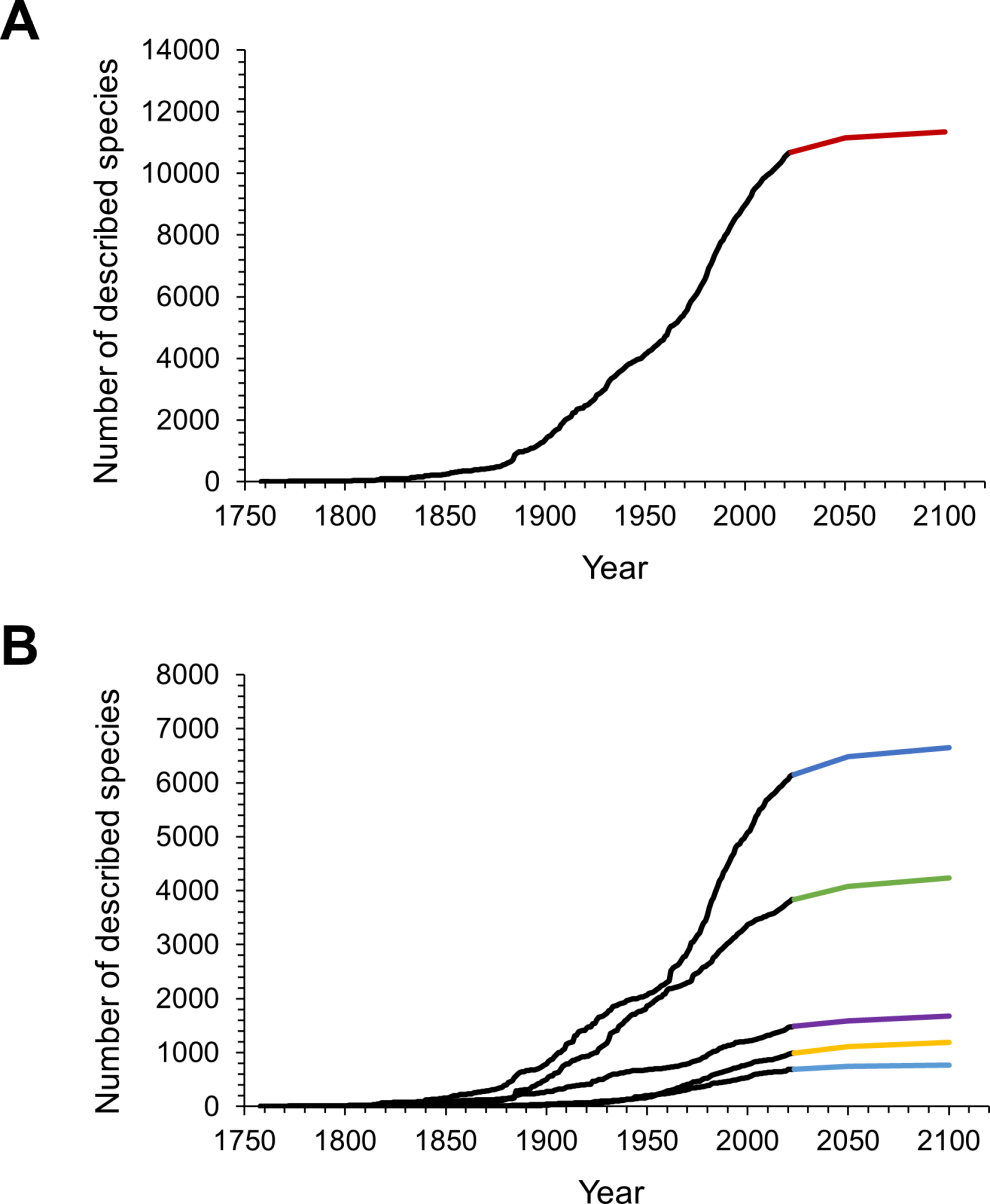
The observed and predicted cumulative number of isopod species described over time. (A) Observed (black line) and predicted (red line) cumulative number of all isopod species. (B) Observed (black lines) and predicted cumulative numbers of species within the subgroups (dark blue: marine; green: terrestrial; purple: parasitic; yellow: subterranean and light blue: freshwater isopods).

The NHRP model predicted another 470 isopod species to be described by the year 2050 with a 95% confidence interval of 390 to 560 (Fig. 3A). Until 2100 a total of 660 (540–810) species were predicted to await scientific description, assuming the pace of description continues at its current rate. This would bring the cumulative number of isopod species up to 11,347 in 2100 (Fig. 3A). When split into subgroups, estimates from the model show that most of the future discoveries could be expected in marine and terrestrial environments, and only a small part will be from freshwaters (Fig. 3B).

### Taxonomic effort

**Figure 4:**
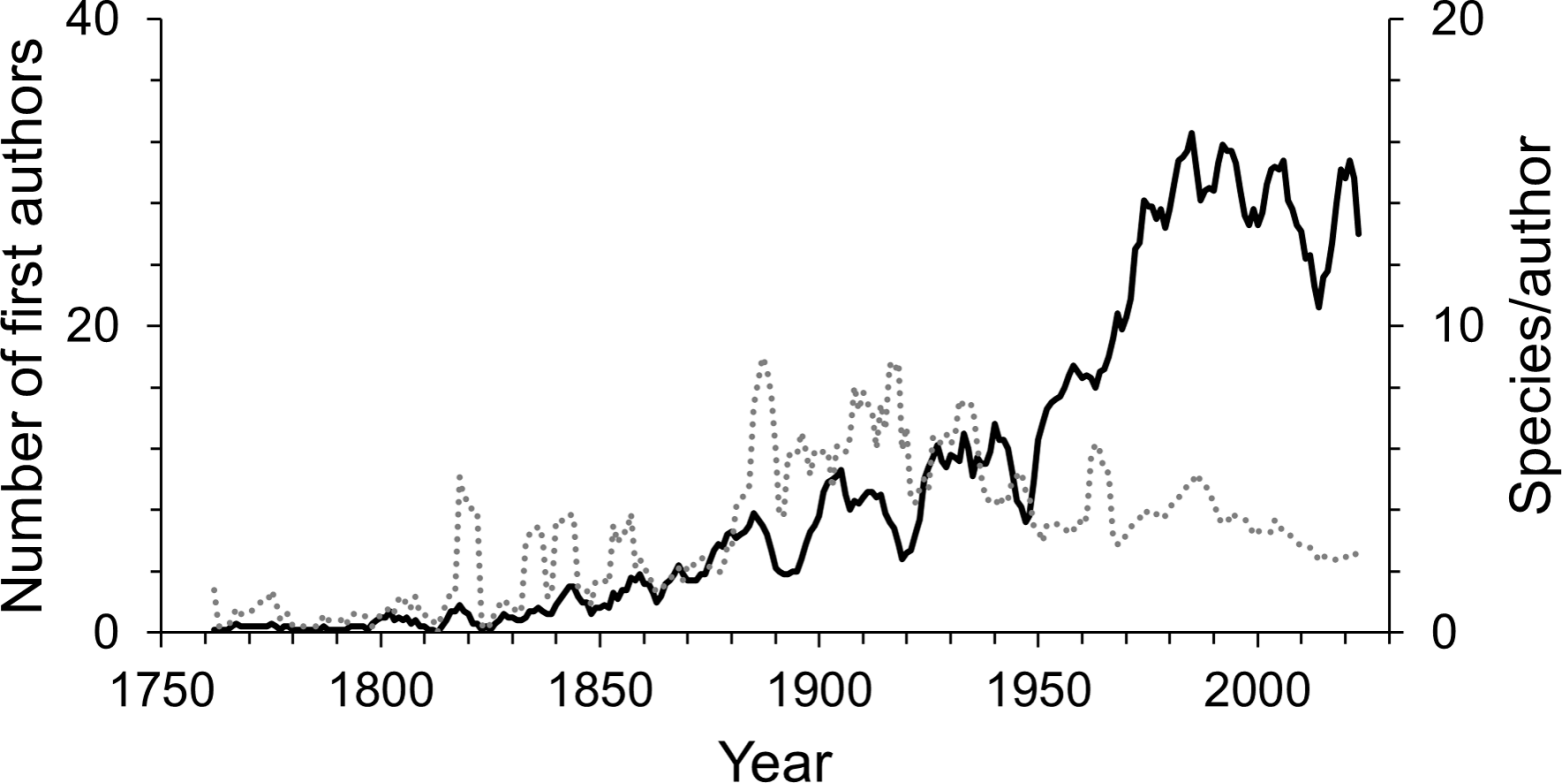
The number of first authors per year (solid line) and the average number of species described per author per year (dotted line). The lines are 5-year moving averages.

**Figure 5:**
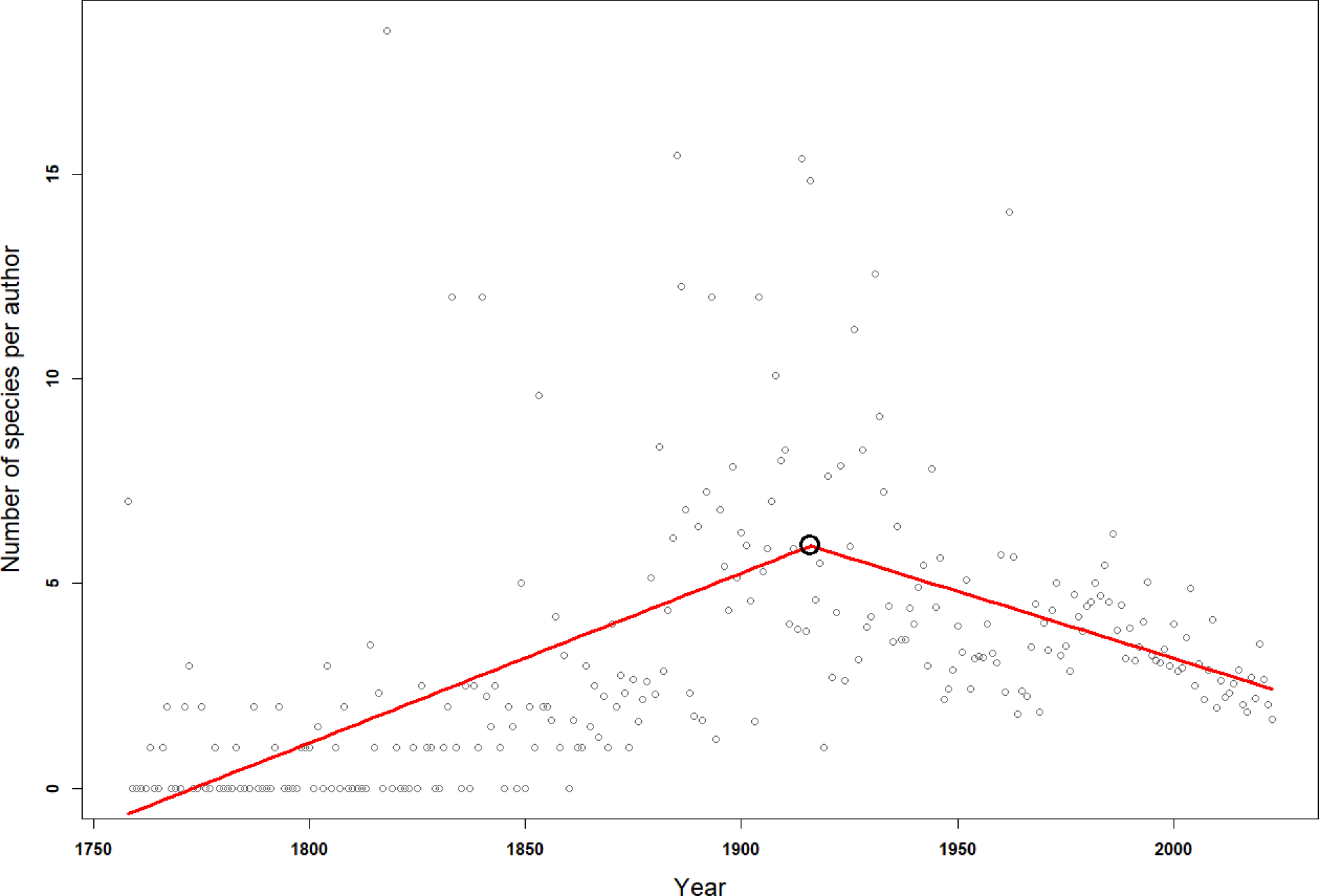
Breakpoint analysis for the average number of species described per number of authors in any given year. The red line is a fitted broken-line of the segmented model. The black circle indicates the breakpoint in 1916.

Since the first scientific description of an isopod species by Linnaeus, 755 first authors have described the species known today. Over time the number of first authors per year has increased. Since the 1950s there were more than three times as many authors involved in isopod taxonomy as during the first half of the 20th century (Fig. 4). This pattern can be seen in almost all subgroups (Fig. S1). However, the average number of species described per author has been declining over the last century (Fig. 4). Nevertheless, the overall trend sees many more taxonomists describing fewer species. A piecewise regression analysis found the breakpoint in the data series to be in 1916, whether zero values were excluded or not (Fig. 5). Since then, the average number of species described per authors active in the same year has declined.

The average publication lifetime of an author was found to be 8.4 years, with 30% of authors ranking above the average. Although a linear regression shows a weak decreasing trend (*R*^2^ = 0.006, *P* < 0.05) in publication lifetime over the years (Fig. S2A), this change was not significant (*R*^2^ = 0.00004, *P* = 0.88) when data of authors who started publishing after 2010 were excluded (Fig. S2C) because these authors may still be publishing in the future. Again, a weak decreasing trend of publication lifetime (*R*^2^ = 0.01, *P* < 0.05) could be detected when all one-time authors were excluded from the linear regression analysis (Fig. S2B), but this trend was again not significant (*R*^2^ = 0.0002, *P* = 0.77) when data for authors who started publishing after 2010 were also excluded (Fig. S2D). Furthermore, there was no significant evidence (*P* > 0.05) for a change in productivity over time, whether Vanhöffen was included (Fig. S3A) or excluded (Fig. S3B).

**Figure 6:**
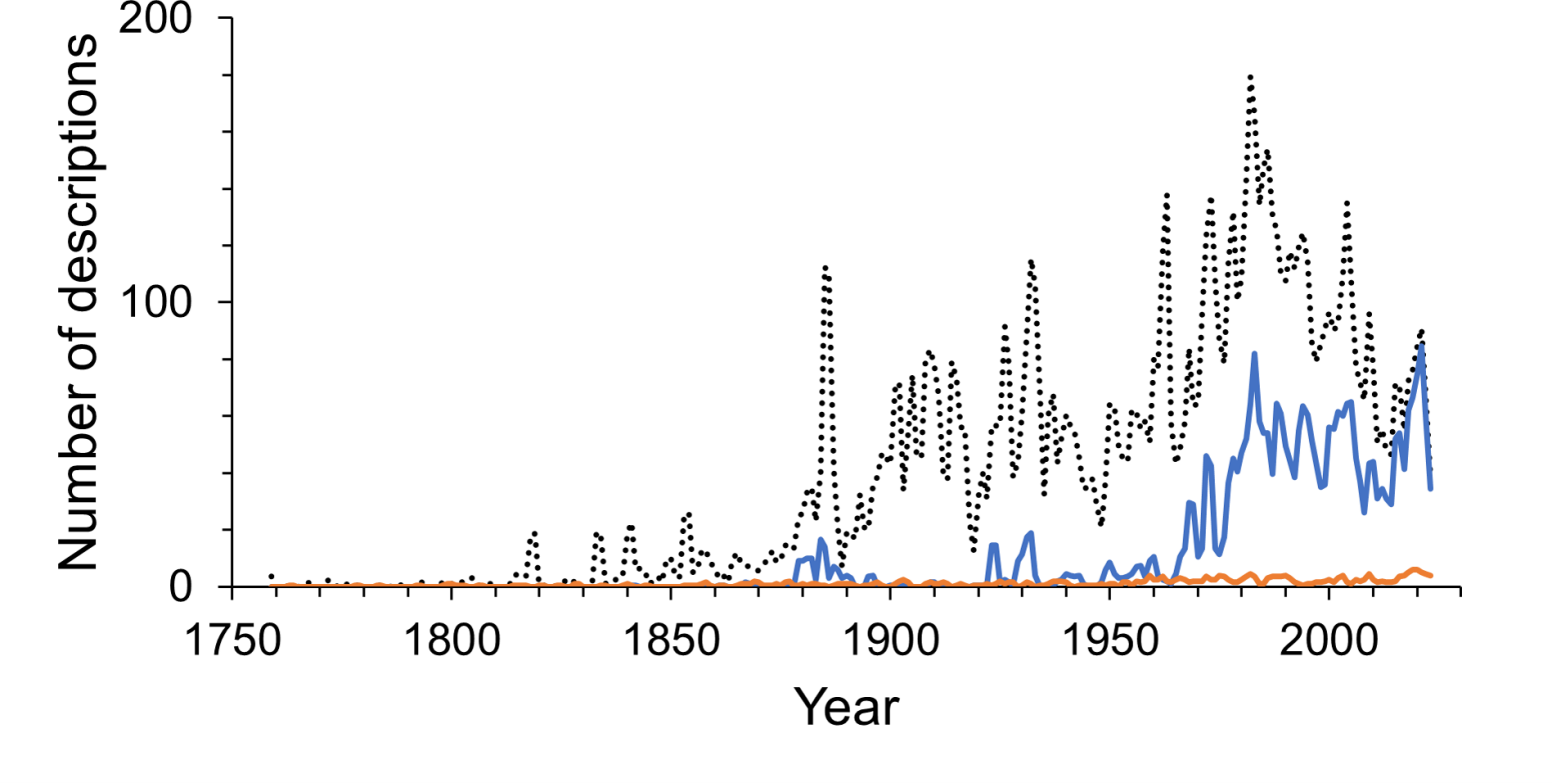
Annual number of multi-authored isopod descriptions and contributions by one-time authors. The dotted line shows the number of all species descriptions. The solid blue line shows the multi-authored contributions per year and the solid orange line shows the number of descriptions made by one-time authors. The lines are 2-year moving averages.

**Figure 7:**
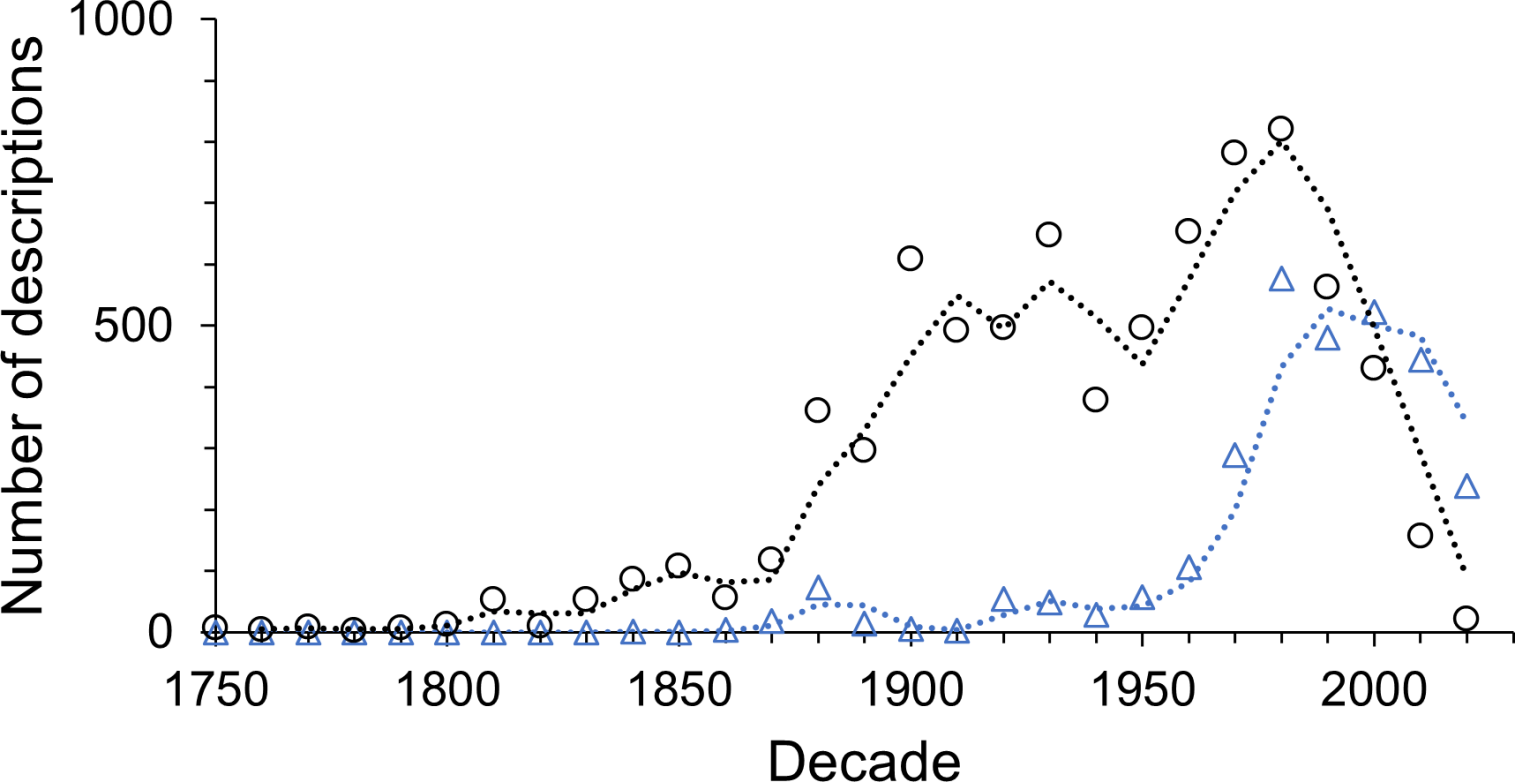
The number of descriptions published by sole (black circles) and multiple authors (blue triangles) in each decade.

Multi-authored descriptions became more abundant during the late 19th century but stayed relatively low until the late 1960s (Fig. 6). Since the beginning of the 21st century multi-authored descriptions outnumbered the number of species described by a sole author (Fig. 7), peaking at a proportion of about 70% of new species descriptions during the 2010s and slightly over 90% within the first three years of the current decade (Fig. S4B). In contrast, the number of descriptions published by one-time authors is negligible (Fig. 6). Their proportions were high in the early history of isopod discovery (Fig. S4A) when the overall number of descriptions was low. However, since the late 19th century, the contribution of one-time authors to isopod taxonomy has been small. During this time span, the highest proportion of one-time authors was found in the current decade with close to 7% (Fig. S4A). In the last “full” decade , the 2010s, the proportion of descriptions by one-time authors was about 5%.

## Discussion

### Named and unnamed species diversity

A decrease in the annual number of species described started more than three decades ago for all isopod species. Because this trend is not a short-term one, it cannot be explained by a time lag in data entry into the database. Estimates for future descriptions of species new to science from the non-homogeneous renewal process model predict approximately 660 additional species to be described until 2100. This suggests that 94% of isopod species that are predicted to be named by the end of this century already have been described. For other groups it has been estimated that about two thirds of all species are described, including stoneflies (DeWalt & Ower, 2019), scale insects (Deng et al., 2016), polychaete worms (Pamungkas et al., 2019), amphipods (Arfianti, Wilson & Costello, 2018) and the world’s marine species in general (Costello, Wilson & Houlding, 2012). Bryozoans have been labeled “one of the better-known taxa on Earth” due to the fact that about 80% of species predicted to be named by 2100 already had been described (Pagès-Escolà et al., 2020). Therefore, isopods represent a very well-known taxon. Of course, as more data will become available in the future these predictions may change. Bebber et al. (2007) showed that unless a taxon’s species inventory is at least 90% complete extrapolations based on existing data may be associated with large margins of error.

Some more conspicuous taxa showed a decline in new species descriptions many decades ago, *i.e.,* mammals globally (Fisher et al., 2018) and birds and flowering plants in the UK (Bebber et al., 2007). An asymptote in description rates was reached about one hundred years ago in well-studied regions, notably in Europe for mammals, birds, black corals, echiurans and euphasiid crustaceans (Wilson & Costello, 2005), as well as for fish, gastropods, sponges, cnidarians, echinoderms, bryozoans and tunicates in Britain and Ireland (Costello, Emblow & Picton, 1996). Although an asymptote has not yet been shown for taxa globally, our data suggest that it may be emerging for isopods. Time will provide the confirmation needed. Similar analyses to those presented here for other taxa may show them to be reaching an asymptote as well.

This study did not take into account the number of already discovered but not yet formally described isopod species deposited in museum and research collections. Fontaine, Perrard & Bouchet (2012) noted an average shelf life of 21 years between the discovery and the taxonomic description of a new species. However, they also found that aquatic species have a shorter shelf life than terrestrial ones and that the shelf life of newly discovered invertebrate species is shorter than for plant or vertebrate species. For recently described isopod species, shelf life varied between 0 years (Monticelli Cardoso, Bastos-Pereira & Lopes Ferreira, 2022) and 54 years (Williams, Boyko & Marin, 2020) with an overall tendency toward the lower range of the spectrum. For example, Malek-Hosseini et al. (2022) described a new groundwater species from Iran within three years of sampling, additionally using molecular data to corroborate its species status. In contrast, the material from which the first bopyrid isopod species from hydrothermal vents was described was collected 21 to 10 years before its taxonomic description (Kato et al., 2022). Naturally, field sampling continues to unearth new species. Depending on the sampling location, the proportions of reported unnamed isopod species in field samples may vary from none (in historically well-studied areas like Europe) to about 18% (López-Orozco et al., 2022 identified three new terrestrial species) and up to as much as 93% (Poore et al., 2015 found that only 9 of 127 marine species from western Australia were previously known to science). From the latter study, none of the sampled species were identifiable with any of the 359 isopod species collected on the continental slope of south-eastern Australia of which 90% were undescribed at the time of sampling (Poore, Just & Cohen, 1994), making Australia a rich source of new isopod species. Similarly, Brandt et al. (2007) found that only 13% of the discriminated 674 deep-sea isopod species from Southern Ocean samples were known to science. Thus, the Southern Ocean as well as the waters around Australia may account for a high proportion of the yet undescribed species globally. However, when these species will be described remains unknown. A list of 21 studies which reported undescribed species (Table S2) contains 1,225 possible new isopod species, of which most were sampled in the deep sea and in and around Australia. Given the average description rate of 75 descriptions/year from the past 20 years, it would take about 16 years to formally describe all these species. It has to be noted that those species were undescribed at the time of publication of the respective study. It has not been checked whether any of the reported species have been formally described since and might now already be part of our dataset of globally described isopod species. However, it is encouraging that a significant proportion of yet-to-be described species may already be collected and awaiting description.

Although scientists are continuously adding new names to the isopod inventory, not all of those names will prove to be valid. Several newly described species might be placed into synonymy over the years. Bouchet (2006) suggested that 10–20% of new species described each year will turn out to be synonyms. Likewise, Appeltans et al. (2012) note that it takes time to discover synonyms and estimated that for every five newly named species, at least two had already been described. Most synonymies will likely be identified and resolved during comprehensive revisions of isopod genera or families (*e.g.*, Stransky et al., 2020; Taiti & Monticelli Cardoso, 2020). Examination of museum specimens may reveal synonyms (Hughes, Bruce & Osborn, 2020), as well as lead to the recognition of species new to science (Garcia, 2020). Thus, taxonomic revisions can decrease the number of accepted species, as well as discover new species.

### Cryptic diversity

Another issue that adds to uncertainty about the number of existing isopod species is cryptic diversity whereby species can only be distinguished using molecular methods. However, isopod and other crustacean taxonomists stated they could always find morphological differences on close examination and thus true cryptic diversity in isopods is negligible (Appeltans et al., 2012, Supplemental Information). Recent years have seen an increase in species delimitation studies using molecular data as well as integrative taxonomic approaches (Pante, Schoelinck & Puillandre, 2015), with some of them discovering putative new species. Species under scrutiny in such cryptic diversity studies tend to be geographically widespread species either in the deep sea (Raupach et al., 2007) or coastal habitats (Hurtado et al., 2016) or recognised species complexes already thought to harbour hidden diversity (Schnurr et al., 2018). Held (2003), for instance, tested the single-widespread-species-hypothesis of a morphologically variable Antarctic serolid isopod and identified two strongly distinct genetic clades uncovering an overlooked species. Likewise, a molecular analysis by Schnurr et al. (2018) disentangled two widely distributed munnopsid species complexes in Icelandic waters. Their data suggested that the *Eurycope producta* species complex consists of eight separate species, and the *Eurycope inermis* complex harbours four distinct species. Some of the discovered genetic clades could be linked to other already described species, leaving a total of seven species new to science. Even more putative new species have been uncovered during a genetic study of *Haloniscus* species from groundwater, springs, caves and salt lakes in Australia (Guzik et al., 2019). Each of the 26 new species was found to be restricted to a small geographical range. However, almost none of the previously unknown species detected by genetic sampling were truly cryptic species. Morphological characters could be found in just about every case, separating the new species from similar ones. Circling back to the problem of collected but unnamed species, few of the newly delimited species from molecular studies were formally described following their detection (Schlick-Steiner et al., 2007; Pante, Schoelinck & Puillandre, 2015). Most studies note that additional taxonomic work is required to fully support a species hypothesis with a combination of DNA data and morphological characters (*e.g.*, Guzik et al., 2019; Jennings, Golovan & Brix, 2020). While molecular methods can be helpful in indicating specimens which may represent new species, and have been used since the 1980s for isopods and other taxa, there is no indication that they significantly increase description rates overall (Appeltans et al., 2012).

### Taxonomic effort

The number of taxonomists describing new species of isopods has increased markedly over time, as it has for all taxa globally. Over the past fifty years, more authors have described isopod species than ever before (Fig. 4). Only for authors describing freshwater isopods has there been a steep decline within the past two decades (Fig. S1B), and this substantial decline is also evident in species numbers. Although it seems that freshwaters may not yield many more new species, it has been suggested that nonsaline environments harbour high cryptic diversity (Wilson, 2008). Indeed, a meta-analysis of cryptic diversity studies found that more posited cryptic species have been discovered in freshwater than in terrestrial or marine environments (Poulin & Pérez-Ponce de León, 2017). However, whether this genetic diversity translates into high species diversity is uncertain. Another interpretation of the decline in new freshwater species could be less taxonomic interest, but there seems no reason to assume why this may be the case.

Increasing numbers of people describing new species have been found in all similar studies (*e.g.*, Joppa, Roberts & Pimm, 2011a; Appeltans et al., 2012; Tancoigne & Dubois, 2013; Costello, Vanhoorne & Appeltans, 2015; Arfianti, Wilson & Costello, 2018; Pamungkas et al., 2019; Pagès-Escolà et al., 2020), at least partly contradicting a not uncommon view that the field of taxonomy is in crisis (Godfray, 2002; Hopkins & Freckleton, 2002; Bacher, 2012). There is no doubt that taxonomy will benefit from more funding and renewed prestige (Agnarsson & Kuntner, 2007; Christenhusz & Byng, 2016; Higgs, 2016), but a lack of people describing new species is not evident from the data. The field of taxonomy is not in decline but changing. It modernised itself from a primarily morphological discipline towards a multi-disciplinary field including genetics and phylogeny. Integration of these different skill sets could explain the now higher number of multi-authored descriptions. To avoid this trend of increasing proportions of multi-authored descriptions from affecting the trend in numbers of active taxonomists over time, only the first author of a species description was considered in our analysis. Therefore, the given numbers of authors contributing to isopod taxonomy are an underestimate of the taxonomic force. Also, the proportion of authors who described only a single isopod species has not increased for more than a century. Nor have taxonomists’ publication lifetimes significantly decreased over this time. This further indicates that the increased number of taxonomic authors is an increase in effort, as concluded by others on other taxa (Joppa, Roberts & Pimm, 2011b; Appeltans et al., 2012; Essl et al., 2013), and not reduced by having proportionally more part-time taxonomists or more people who stop publishing descriptions after only a few years.

The present analysis did not consider the level of expertise of every author because this could not be determined from the available data. Some are well-established taxonomists who have spent a lifetime building up their extensive knowledge of a taxon and can therefore be considered true experts. Others are at the start of their career and still working towards expert status. Again, others contribute an essential amount of their work in other research fields, nevertheless adding valuable information with every published species description. Some people do not think it appropriate to call everyone who describes a species a taxonomist (Wheeler, 2014) and most likely, not everyone who does describe a species now and then would characterise themselves as such. However, regardless of which labels one puts on the authors of species descriptions, the fact remains that all of them contribute to the scientific inventory of the planet’s biodiversity and draft testable hypotheses. Our data show that the percentage of people who publish only a single species description is tiny and has not increased for over a century. For more information on the perceived and detectable loss of expertise and the state of taxonomy in different countries, see Lovejoy et al. (2010), Boxshall & Self (2011) and Coleman (2015), and the Australian Academy of Science (Taxonomy Decadal Plan Working Group, 2018). These assessments of taxonomy in the UK, Canada and Australia and New Zealand all considered people who described new species as a sub-set of all those working in taxonomy.

Although there have never been so many taxonomic authors than in recent decades, the average annual number of isopod species described per taxonomist has declined strongly over the last century. Such a decline in species per taxonomist has also been found for the closely related Amphipoda (Arfianti, Wilson & Costello, 2018) and for other taxa, such as scale insects (Deng et al., 2016), flowering plants (Joppa, Roberts & Pimm, 2011a), as well as spiders, amphibians, birds and mammals (Joppa, Roberts & Pimm, 2011b), marine and terrestrial parasites (Costello, 2016), fossil and extant marine bryozoans (Pagès-Escolà et al., 2020) and overall marine and non-marine species (Costello, Wilson & Houlding, 2013). The reduction in the description rate of isopod species observed here, despite peak numbers of taxonomists, suggests that most species have already been named, as concluded for other taxa (Joppa, Roberts & Pimm, 2011b; Arfianti, Wilson & Costello, 2018; Pamungkas et al., 2019). Contradicting this interpretation, Sangster & Luksenburg (2015) proposed that the lower number of species described per taxonomist is rather a consequence of the improved quality of species descriptions than a slowdown of progress in species discovery. They found that the number of pages of taxonomic descriptions has increased compared to the 1930s. So has the number of specimens on which the description of a new species is based, the number of characters to differentiate it from its most closely related species and the number of illustrations in a publication. With this increased effort put into the scientific description of a species, it may take more time from the initial discovery of a species until the publication of its formal description. However, other studies point to greater efficiencies in taxonomy due to greater access to field samples and literature, and improved museum collections, laboratory methods, publication efficiency, and communication between people (Eschmeyer et al., 2010; Costello, Houlding & Wilson, 2014). We found a similar productivity of taxonomists over their isopod-description careers, indicating that modern efficiencies and co-authorships may indeed balance out the richer species descriptions.

At the upper end of productivity, 21 taxonomists (only 3% of the taxonomic workforce over time) have described approximately 43% of all known isopod species. The three most prolific authors described almost exclusively terrestrial isopod species, which are more easily accessible and can be sampled without the deployment of advanced sampling equipment by comparison with marine isopods. Accordingly, our model estimates suggest that a considerable proportion of future discoveries might be made in the less accessible marine environment. Also, because large and geographically widespread species tend to be named first (Costello et al., 2015; Higgs & Attrill, 2015), many of the yet-undiscovered isopod species are likely to be small and/or geographically restricted species (Scheffers et al., 2012; Liu et al., 2022). There is speculation on whether most of the yet-undescribed species will be found in collections (Scheffers et al., 2012; Coleman, 2015) or will be newly discovered during fieldwork (Grieneisen et al., 2014). However, both named and unnamed species, especially freshwater and endemic species, are at risk of extinction due to human impacts (Costello, 2015; Liu et al., 2022). Because many new species tend to be discovered in biodiversity rich-spots, which already face many threats like extensive habitat loss, they will be more vulnerable (Scheffers et al., 2012; Manes et al., 2021) and are at risk of going extinct before they are even discovered (Costello, May & Stork, 2013). It is therefore important that taxonomists continue to describe new species. Only named, and as such well delimited species, can be included in threat reports and conservation plans.

## Conclusions

Considerable progress has been made in the description of isopod species, with 696 freshwater, 3,840 terrestrial and 6,151 marine species named by 2023, of which 994 species are categorised as subterranean and 1,486 as parasitic. Descriptions peaked in the 1980s and have been declining in all groups. They have been supported by an increasing number of taxonomists since the 19th century. However, the number of species described per taxonomic effort has been declining since 1916. Using a statistical model, we estimated that approximately 660 additional isopod species will be described by 2100. The more data become available in the future, the more accurate estimated species numbers will become and the closer those estimates will get to the real total. Taxonomists have already named and described a substantial proportion of the world’s isopod species and our data raise the hope that the completion of a global isopod inventory is an achievable task.

##  Supplemental Information

10.7717/peerj.15984/supp-1 Table S1 A list of the most prolific authors (who described each more than 100 isopod species –as first authors) Together they described 4,619 species, approximately 43% of the total.

10.7717/peerj.15984/supp-2 Table S2 A selection of 21 studies (selected by chance) which found and reported undescribed isopod species Some studies described a few of the collected unnamed species right away, so the numbers given in the table below are the number of species that were left undescribed. Note that those species were undescribed at the time of publication of the respective study. It has not been checked whether any of the reported species have been formally described since and might now already be part of our dataset of globally described isopod species.

10.7717/peerj.15984/supp-3 Figure S1 The number of first authors per year (solid line) and the average number of species described per author per year (dotted line) for the various subgroups (A) marine, (B) freshwater, (C) terrestrial, (D) parasitic and (E) subterranean. The lines are 5-year moving averages. Note that the scales vary.

10.7717/peerj.15984/supp-4 Figure S2 Linear regressions of authors’ publication lifetimes against the year of the first publication (start of their publication lifetime) (A) for all first authors, (B) one-time authors excluded, (C) for all first authors, excluding the ones, who started publishing after 2010, (D) one-time authors and first authors, who started publishing after 2010, excluded.

10.7717/peerj.15984/supp-5 Figure S3 Linear regressions of publication lifetime against the average yearly number of species described by each author (A) all first authors, (B) Vanhöffen, who described 67 species in a single year, excluded.

10.7717/peerj.15984/supp-6 Figure S4 The percentage of contributions (A) by one-time authors, who described only a single species and (B) for multi-authored descriptions over time.

## Additional Information and Declarations

### Data Availability

The following information was supplied regarding data availability: The data is available at figshare: Hartebrodt, Lena (2019): World list of isopod species and their authorities.xlsx. The University of Auckland. Dataset. https://doi.org/10.17608/k6.auckland.9927278.v3. Hartebrodt, Lena (2023). World list of isopod species and their authorities_updated_2023.xlsx. The University of Auckland. Dataset. https://doi.org/10.17608/k6.auckland.23258675.v1.
