# Supplementary material for: Progress in the discovery of isopods (Crustacea: Peracarida)—is the description rate slowing down?
Source: PeerJ. 2023 Sep 4;11:e15984. doi: 10.7717/peerj.15984 (PMC10484202; doi:10.7717/peerj.15984)
Supplement: Table S1 — Together they described 4,619 species, approximately 43% of the total. [file peerj-11-15984-s001.docx]

**Table S1**. A list of the most prolific authors (who described each more than 100 isopod species – as first authors). Together they described 4,619 species, approximately 43% of the total.

| **First author** | **Species described** | | | | **Publication lifetime (years)** | **Species/ year** |
| --- | --- | --- | --- | --- | --- | --- |
|  | Total | Multi-authored | First | Last |  |  |
| Verhoeff | 421 | 4 | 1896 | 1967 | 72 | 6 |
| Vandel | 332 | 0 | 1924 | 1981 | 58 | 6 |
| Budde-Lund | 332 | 0 | 1880 | 1913 | 34 | 10 |
| Bruce, N.L. | 313 | 35 | 1978 | 2022 | 45 | 7 |
| Barnard, K.H. | 305 | 0 | 1914 | 1965 | 52 | 6 |
| Menzies | 302 | 121 | 1950 | 1983 | 34 | 9 |
| Kensley | 289 | 102 | 1971 | 2009 | 39 | 7 |
| Nunomura | 260 | 21 | 1973 | 2019 | 47 | 6 |
| Richardson | 250 | 0 | 1897 | 1913 | 17 | 15 |
| Taiti | 235 | 229 | 1979 | 2020 | 42 | 6 |
| Poore | 211 | 129 | 1975 | 2013 | 39 | 5 |
| Arcangeli | 187 | 0 | 1911 | 1960 | 50 | 4 |
| Ferrara | 178 | 156 | 1971 | 1996 | 26 | 7 |
| Kussakin | 165 | 64 | 1955 | 2001 | 47 | 4 |
| Dollfus, A. | 142 | 1 | 1884 | 1905 | 22 | 6 |
| Birstein | 137 | 4 | 1932 | 1972 | 41 | 3 |
| Schmalfuss | 127 | 46 | 1972 | 2016 | 45 | 3 |
| Hansen | 115 | 0 | 1890 | 1916 | 27 | 4 |
| Nierstrasz | 112 | 90 | 1915 | 1941 | 27 | 4 |
| Müller, H.G. | 105 | 3 | 1988 | 1995 | 8 | 13 |
| Schultz | 101 | 5 | 1963 | 1995 | 33 | 3 |
