# Supplementary material for: Progress in the discovery of isopods (Crustacea: Peracarida)—is the description rate slowing down?
Source: PeerJ. 2023 Sep 4;11:e15984. doi: 10.7717/peerj.15984 (PMC10484202; doi:10.7717/peerj.15984)
Supplement: Table S2 — Some studies described a few of the collected unnamed species right away, so the numbers given in the table below are the number of species that were left undescribed. Note that those species were undescribed at the time of publication of the respective study. It has not been checked whether any of the reported species have been formally described since and might now already be part of our dataset of globally described isopod species. [file peerj-11-15984-s002.docx]

**Table S2**. A selection of 21 studies (selected by chance) which found and reported undescribed isopod species. Some studies described a few of the collected unnamed species right away, so the numbers given in the table below are the number of species that were left undescribed. Note that those species were undescribed at the time of publication of the respective study. It has not been checked whether any of the reported species have been formally described since and might now already be part of our dataset of globally described isopod species.

| **Study** | **Location** | **# collected but undescribed species** |
| --- | --- | --- |
| Brandt et al., 2007 | Southern Ocean, deep sea | 585 |
| Poore et al., 1994 | South-eastern Australian continental slope | 318 |
| Poore et al., 2015 | Western and south-western Australian continental margin | 118 |
| Wilson, 2008 | Gulf of Mexico, deep sea | 60 |
| Jennings et al., 2020 (genetic study) | Kuril-Kamchatka trench, deep sea | 34 |
| Guzik et al., 2019 (genetic study) | Australia, terrestrial | 26 |
| Golovan, 2018 | Kuril Basin, deep sea | 19 |
| Kavanagh, 2009 | Ireland, deep sea | 15 |
| Wetzer et al., 1997 | Santa Maria Basin and Western Santa Barbara Channel | 14 |
| del Carmen Espinosa-Pérez & Hendrickx, 2001 | Eastern Tropical Pacific | 8 |
| Schnurr et al., 2018 (genetic study) | Transition zone btw. North Atlantic and Nordic Seas | 7 |
| Bunkley-Williams et al., 2006 | Venezuela, marine | Potentially up to 4 |
| Graening & Rogers, 2013 | California, terrestrial | Potentially up to 4 |
| Bluhm et al., 2005 | Canada Basin, deep sea | 3 |
| Boyko & Williams, 2004 | Bahamas, shallow marine waters | 2 |
| Merrin, 2006 | Tasman Sea | 2 |
| Magrini et al., 2010 | Brazil, terrestrial | 1 or 2 |
| Held, 2003 (genetic study) | Antarctic, shallow marine waters | 1 |
| Brockerhoff, 2004 | New Zealand, intertidal | 1 |
| Xavier et al., 2012 (genetic study) | North-east Atlantic and Mediterranean | 1 |
| Golovan & Malyutina, 2022 | Bering Sea, deep sea | 1 |

**References**

Bluhm, B. A., MacDonald, I. R., Debenham, C., & Iken, K. (2005). Macro- and megabenthic communities in the high Arctic Canada Basin: Initial findings. *Polar Biology*, *28*(3), 218–231.

Boyko, C. B., & Williams, J. D. (2004). New records of marine isopods (Crustacea: Peracarida) from the Bahamas, with descriptions of two new species of epicarideans. *Bulletin of Marine Science*, *74*(2), 353–383.

Brandt, A., Brix, S., Brökeland, W., Choudhury, M., Kaiser, S., & Malyutina, M. (2007). Deep-sea isopod biodiversity, abundance, and endemism in the Atlantic sector of the Southern Ocean — Results from the ANDEEP I–III expeditions. *Deep Sea Research Part II: Topical Studies in Oceanography*, *54*(16–17), 1760–1775. https://doi.org/10.1016/J.DSR2.2007.07.015

Brockerhoff, A. M. (2004). Occurrence of the internal parasite *Portunion* sp. (Isopoda: Entoniscidae) and its effect on reproduction in intertidal crabs (Decapoda: Grapsidae) from New Zealand. *Journal of Parasitology*, *90*(6), 1338–1344.

Bunkley-Williams, L., Williams, E. H., & Bashirullah, A. K. (2006). Isopods (Isopoda: Aegidae, Cymothoidae, Gnathiidae) associated with Venezuelan marine fishes (Elasmobranchii, Actinopterygii). *Revista de Biología Tropical*, *54*, 175–188.

del Carmen Espinosa-Pérez, M., & Hendrickx, M. E. (2001). Checklist of isopods (Crustacea: Peracarida: Isopoda) from the eastern tropical Pacific. *Belgian Journal of Zoology*, *131*(1), 43–56.

Golovan, O. A. (2018). Desmosomatidae (Isopoda: Asellota) from the Kuril Basin of the Sea of Okhotsk: First data on diversity with the description of the dominant species *Mirabilicoxa biramosa* sp. nov. *Deep Sea Research Part II: Topical Studies in Oceanography*, *154*, 292–307. https://doi.org/10.1016/J.DSR2.2018.01.008

Golovan, O. A., & Malyutina, M. (2022). The first record of the family Paramunnidae (Isopoda: Asellota) from the bathyal of the Bering Sea with descriptions of two new species of *Munnogonium*. *Deep Sea Research Part II: Topical Studies in Oceanography*, *105095*.

Graening, G., & Rogers, D. C. (2013). Checklist of inland aquatic Isopoda (Crustacea: Malacostraca) of California. *California Fish and Game*, *99*(4), 176–192.

Guzik, M. T., Stringer, D. N., Murphy, N. P., Cooper, S. J. B., Taiti, S., King, R. A., Humphreys, W. F., & Austin, A. D. (2019). Molecular phylogenetic analysis of Australian arid-zone oniscidean isopods (Crustacea: *Haloniscus*) reveals strong regional endemicity and new putative species. *Invertebrate Systematics*, *33*(3), 556–574. https://doi.org/10.1071/IS18070

Held, C. (2003). Molecular evidence for cryptic speciation within the widespread Antarctic crustacean *Ceratoserolis trilobitoides* (Crustacea, Isopoda). In *Antarctic Biology in a Global Context*. Backhuys Publishers.

Jennings, R. M., Golovan, O. A., & Brix, S. (2020). Integrative species delimitation of desmosomatid and nannoniscid isopods from the Kuril-Kamchatka trench, with description of a hadal species. *Progress in Oceanography*, *182*, 102236. https://doi.org/10.1016/J.POCEAN.2019.102236

Kavanagh, F. A. (2009). A catalogue of the Asellota (Crustacea: Isopoda) off the west coast of Ireland and Britain, from 100–5000 m. *Bulletin of the Irish Biogeographical Society*, *33*, 14–75.

Magrini, M. J., Araujo, P. B., & Uehara-Prado, M. (2010). Crustacea, Isopoda, Oniscidea Latreille, 1802: New continent record and distribution extension in Brazil. *Check List*, *6*(2), 217–219.

Merrin, K. L. (2006). The first record of the crustacean isopod genus *Pseudarachna* Sars, 1897 (Isopoda: Asellota: Munnopsidae) from the Southern Hemisphere, with description of a new species from New Zealand. *Zootaxa*, *1370*, 59–68. https://doi.org/10.11646/zootaxa.1370.1.5

Poore, G. C. B., Avery, L., Błażewicz-Paszkowycz, M., Browne, J., Bruce, N. L., Gerken, S., Glasby, C. J., Greaves, E., McCallum, A. W., Staples, D., Syme, A., Taylor, J., Walker-Smith, G., Warne, M., Watson, C., Williams, A., Wilson, R. S., & Woolley, S. (2015). Invertebrate diversity of the unexplored marine western margin of Australia: Taxonomy and implications for global biodiversity. *Marine Biodiversity*, *45*(2), 271–286. https://doi.org/10.1007/S12526-014-0255-Y/FIGURES/5

Poore, G. C. B., Just, J., & Cohen, B. F. (1994). Composition and diversity of Crustacea Isopoda of the southeastern Australian continental slope. *Deep Sea Research Part I: Oceanographic Research Papers*, *41*(4), 677–693. https://doi.org/10.1016/0967-0637(94)90049-3

Schnurr, S., Osborn, K. J., Malyutina, M., Jennings, R. M., Brix, S., Driskell, A., Svavarsson, J., & Martinez Arbizu, P. (2018). Hidden diversity in two species complexes of munnopsid isopods (Crustacea) at the transition between the northernmost North Atlantic and the Nordic Seas. *Marine Biodiversity*, *48*(2), 813–843. https://doi.org/10.1007/S12526-018-0877-6

Wetzer, R., Brusca, R. C., & Wilson, G. D. F. (1997). The Crustacea Part 2 - The Isopoda, Cumacea and Tanaidacea. In J. A. Blake & P. H. Scott (Eds.), *Taxonomic Atlas of the Benthic Fauna of the Santa Maria Basin and Western Santa Barbara Channel* (Vol. 11). Santa Barbara Museum of Natural History.

Wilson, G. D. F. (2008). Local and regional species diversity of benthic Isopoda (Crustacea) in the deep Gulf of Mexico. *Deep Sea Research Part II: Topical Studies in Oceanography*, *55*(24–26), 2634–2649.

Xavier, R., Santos, A. M., Harris, D. J., Sezgin, M., Machado, M., & Branco, M. (2012). Phylogenetic analysis of the north‐east Atlantic and Mediterranean species of the genus *Stenosoma* (Isopoda, Valvifera, Idoteidae). *Zoologica Scripta*, *41*(4), 386–399.
