# Supplementary figures and images for: Progress in the discovery of isopods (Crustacea: Peracarida)—is the description rate slowing down?
Source: PeerJ. 2023 Sep 4;11:e15984. doi: 10.7717/peerj.15984 (PMC10484202; doi:10.7717/peerj.15984)

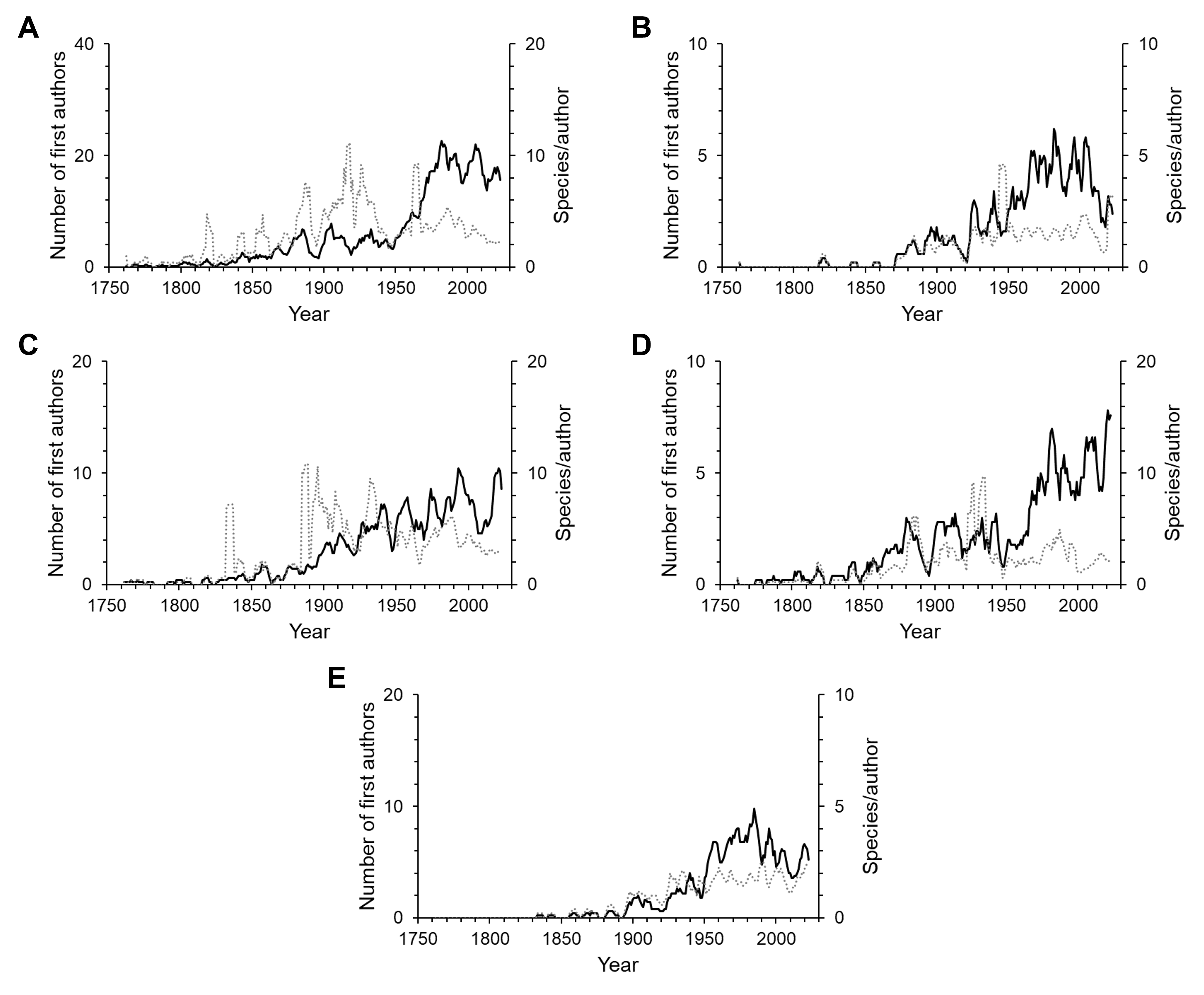

Supplement: Figure S1 — (A) marine, (B) freshwater, (C) terrestrial, (D) parasitic and (E) subterranean. The lines are 5-year moving averages. Note that the scales vary. [file peerj-11-15984-s003.png]

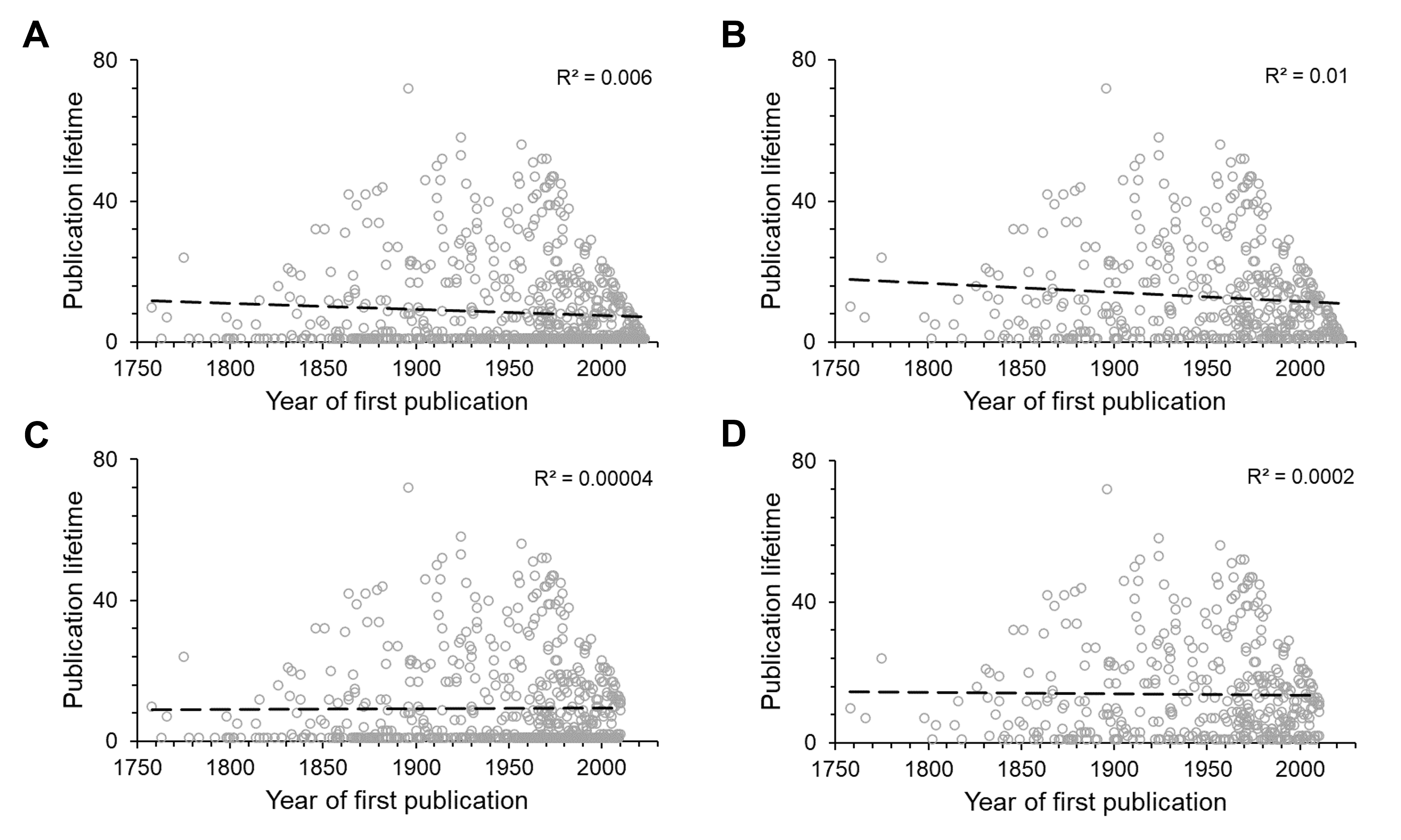

Supplement: Figure S2 — (A) for all first authors, (B) one-time authors excluded, (C) for all first authors, excluding the ones, who started publishing after 2010, (D) one-time authors and first authors, who started publishing after 2010, excluded. [file peerj-11-15984-s004.png]

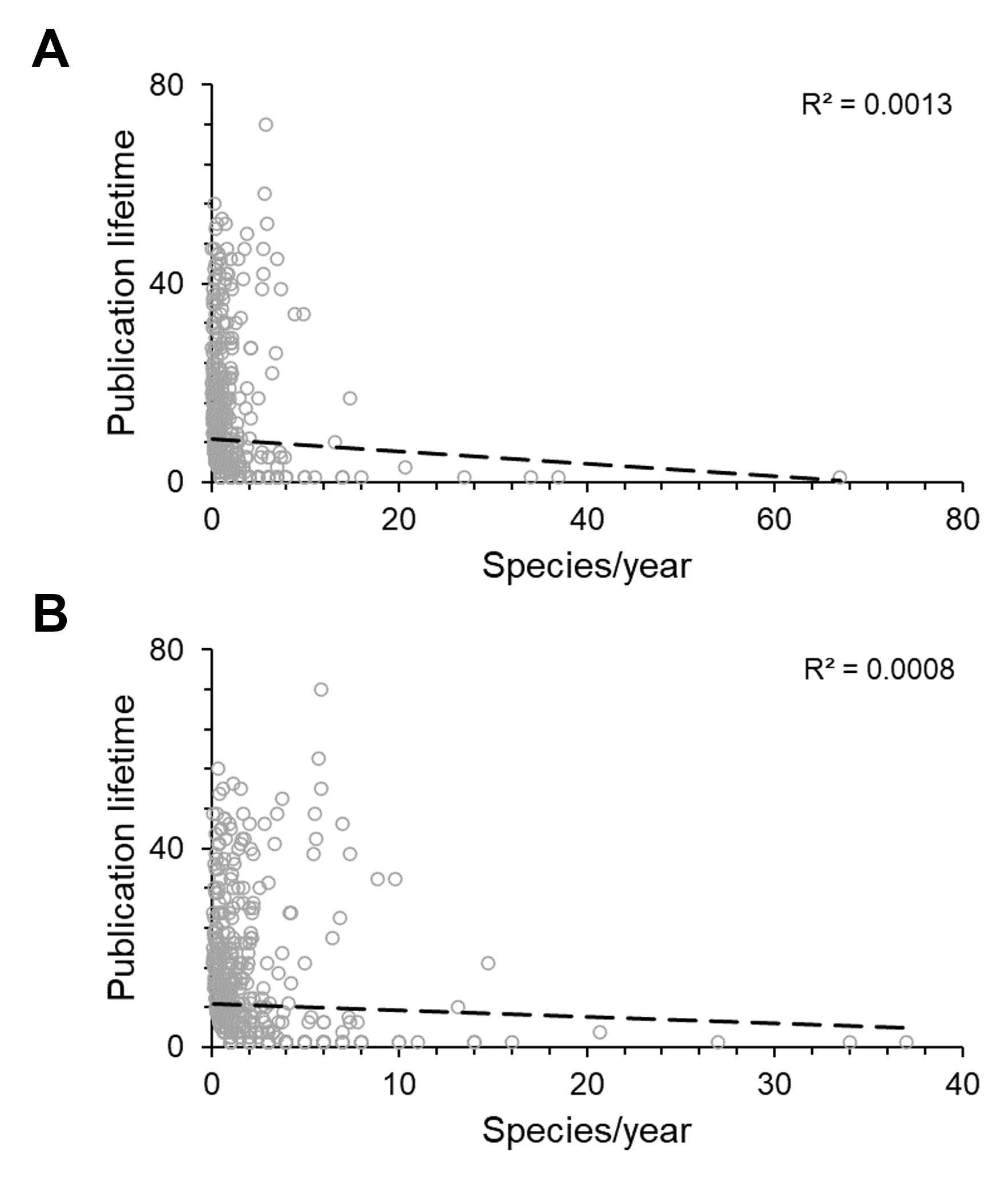

Supplement: Figure S3 — (A) all first authors, (B) Vanhöffen, who described 67 species in a single year, excluded. [file peerj-11-15984-s005.png]

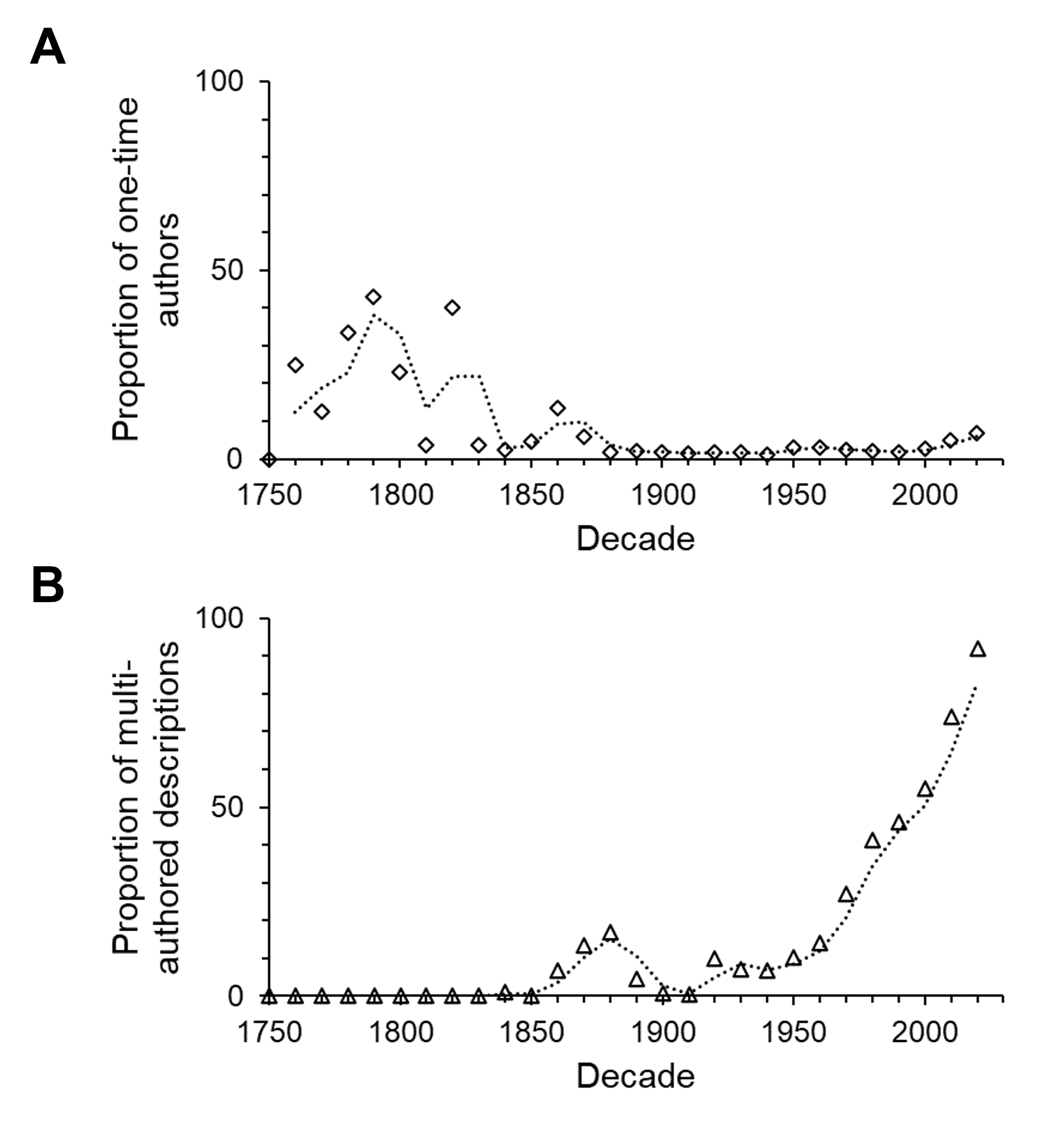

Supplement: Figure S4 — (A) by one-time authors, who described only a single species and (B) for multi-authored descriptions over time. [file peerj-11-15984-s006.png]
